# Delayed Sub-axial Fracture Dislocation Surgical Management: Technical Notes and Review of the Literature

**DOI:** 10.7759/cureus.39539

**Published:** 2023-05-26

**Authors:** Fahad Alhelal, Suhail AlAssiri, Sami I Aleissa, Faisal M Konbaz, Majed Abaalkhail, Husam Altahan

**Affiliations:** 1 Orthopaedic Surgery, King Abdulaziz Medical City Riyadh, Riyadh, SAU; 2 Orthopaedic Surgery, King Abdullah International Medical Research Center, Riyadh, SAU; 3 Orthopaedic Surgery, King Saud Bin Abdulaziz University for Health Sciences, Riyadh, SAU; 4 Orthopaedic Surgery, King Faisal Specialist Hospital and Research Centre, Riyadh, SAU

**Keywords:** vertebral artery injury, quadriplegia, fracture dislocation, cervical vertebrae, delayed sub-axial injuries

## Abstract

The surgical treatment of delayed, unstable sub-axial cervical spine injuries is challenging. Multiple treatment regimens have been described in the literature, although there is no consensus regarding the best treatment approach. This report presents a 35-year-old obese woman who experienced a delayed sub-axial fracture-dislocation following a motor vehicle accident (MVA) and was successfully managed after three weeks via pre-operative traction followed by a novel single-surgery, single-approach technique with pedicle screws and tension-band wiring as a reduction method.

A 35-year-old obese woman with a body mass index (BMI) of 30.1 sustained a frontal impact MVA and suffered from complete quadriplegia below C5 (American Spinal Cord Association Injury A) three weeks prior to presentation. She was intubated and presented with a Glasgow Coma Scale score of 11/15. Trauma computed tomography (CT) showed an isolated spine injury. Moreover, whole-spine CT showed an isolated cervical spine injury involving a basin tip fracture, a comminuted C1 arch fracture, a C2 fracture, and a C6-C7 fracture-dislocation. In addition, magnetic resonance imaging revealed cord contusion at the same level, with C1-C2 left atlantoaxial joint instability. Neck magnetic resonance angiograms and carotid CT angiograms showed left vertebral artery attenuation. She was admitted to the intensive care unit and taken for C6-C7 reduction and instrumentation using only a posterior approach after medical optimization and the application of sufficient traction.

Delayed cervical spine fracture-dislocation imposes a challenge for surgical reduction. However, a proper reduction can be achieved through a sufficient duration of pre-operative traction and an isolated anterior or posterior approach.

## Introduction

Traumatic cervical spine injuries are relatively common. They are estimated to occur in 2-3% of all blunt trauma patients and have a 6% mortality rate and 1% incidence of associated spinal cord injury [1-2]. They can be classified into two main regions: the cranio-cervical junction, which includes the occiput joint to the axis (C2), and the sub-axial region, which consists of C3-C7 [3]. More than half of all cervical spine fractures and dislocations involve the sub-axial cervical spine region and most commonly occur from the fifth to seventh cervical spine levels [1,4].

Furthermore, the vertebral artery (VA) plays a role in injury severity. Fassett et al. reported that 70% of traumatic VA injuries are associated with cervical spine fractures, and the incidence of neurological deficits secondary to VA injuries can reach up to 25% [5-6].

Preoperative planning and imaging and appropriate, timely surgical treatment are essential to achieving satisfactory results. The surgical management of unstable cervical fractures is complex. Late, delayed presentation adds to its complexity and represents a further challenge for spine surgeons, which is why multiple surgical approaches should be considered and individualized on a case-by-case basis [7].

The literature on delayed sub-axial cervical spine injury is relatively limited and infrequently discussed. Thus, we present our single-surgery, single-approach treatment technique for delayed sub-axial cervical spine fracture-dislocation in a middle-aged woman.

## Case presentation

A 35-year-old obese woman (BMI of 30.1) sustained a front-impact motor vehicle accident (MVA) in 2019, three weeks prior to presenting to our hospital. She was sitting in the front seat and was unrestrained. She was intubated and presented with a Glasgow Coma Scale score of 11/15. The patient was reassessed, initially resuscitated, and investigated. A complete neurological exam confirmed she had complete quadriplegia below C5, with Medical Research Council scale grades of 1 in C5 bilaterally and 0 from C6 to S1.

**Figure 1:**
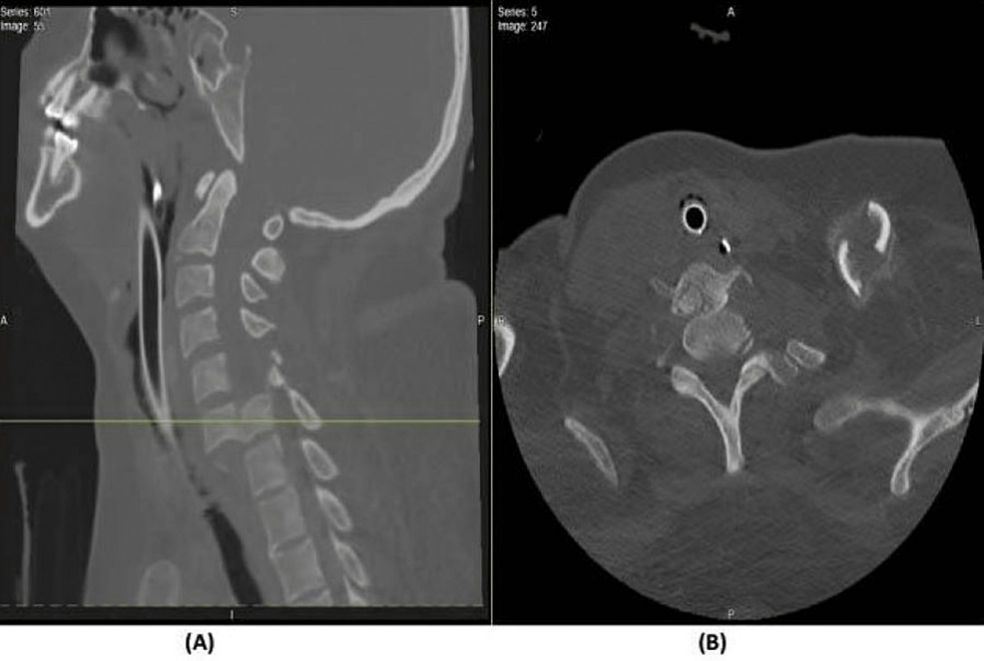
CT scan of C6-7 fracture dislocation (A) Cervical spine CT scan sagittal cuts demonstrating a total fracture dislocation of C6 over C7; (B) cervical spine CT scan axial cuts demonstrating the corresponding level of C6–C7.

**Figure 2:**
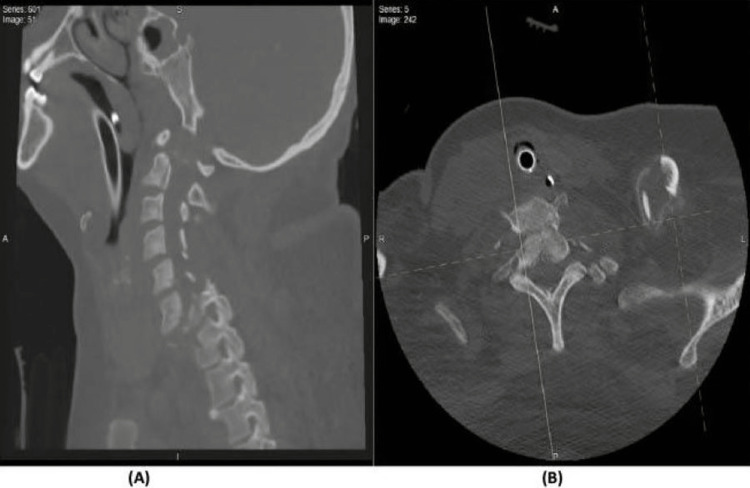
CT scan of C5-6 fracture involvement (A) Cervical spine CT scan sagittal cuts demonstrating a comminuted fracture of C5 transverse process, bilateral pedicles, and spinous process; (B) cervical spine CT scan axial cuts demonstrating the corresponding levels of C5 and C6–C7.

**Figure 3:**
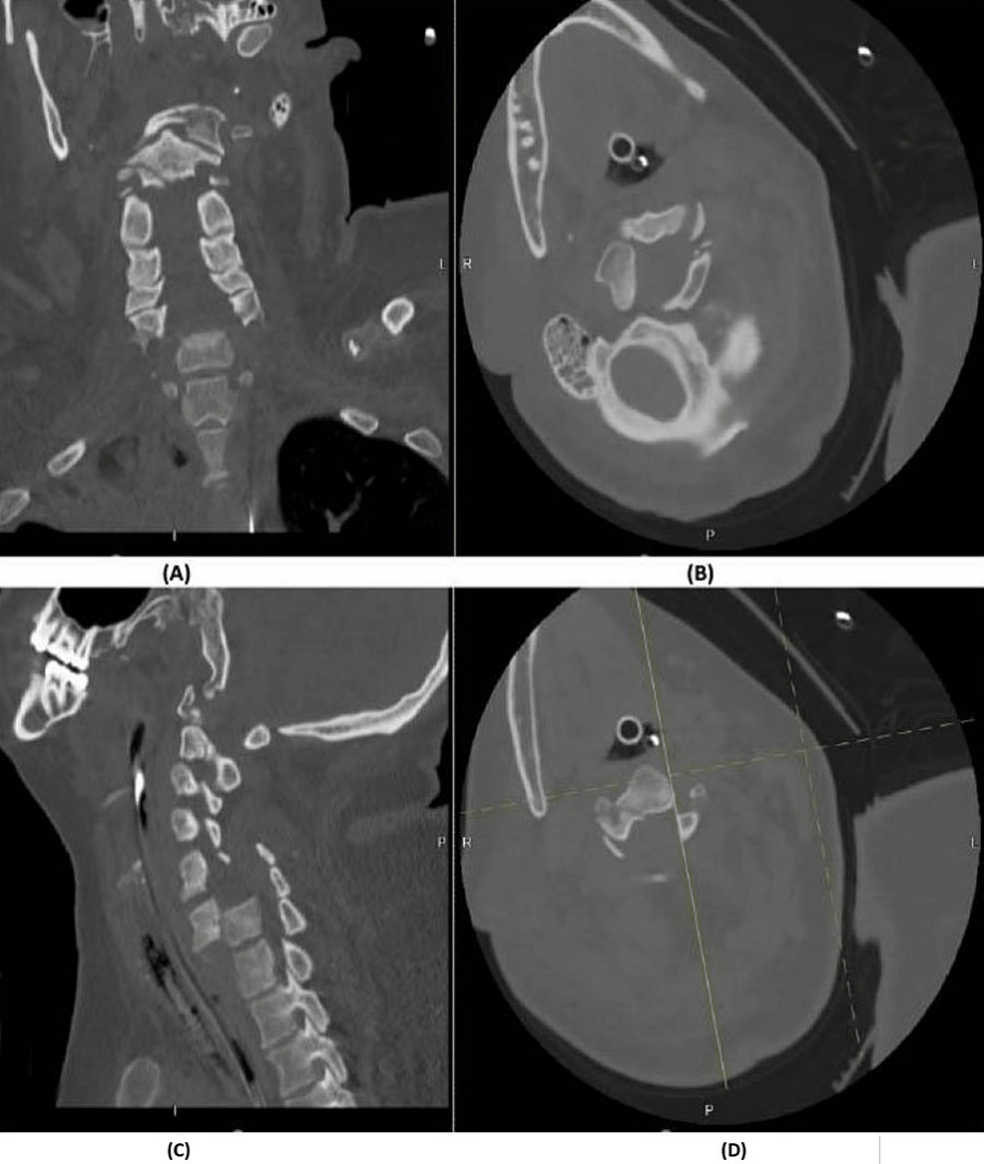
CT scan depicting C2 fracture (A,C) Cervical spine CT scan sagittal and coronal cuts demonstrating a fracture dissecting through the base of C2 and pars interarticularis and bilateral transverse foramina of C2. (B,D) Axial cervical spine CT cuts of the corresponding level of C1–C2 vertebral body.

**Figure 4:**
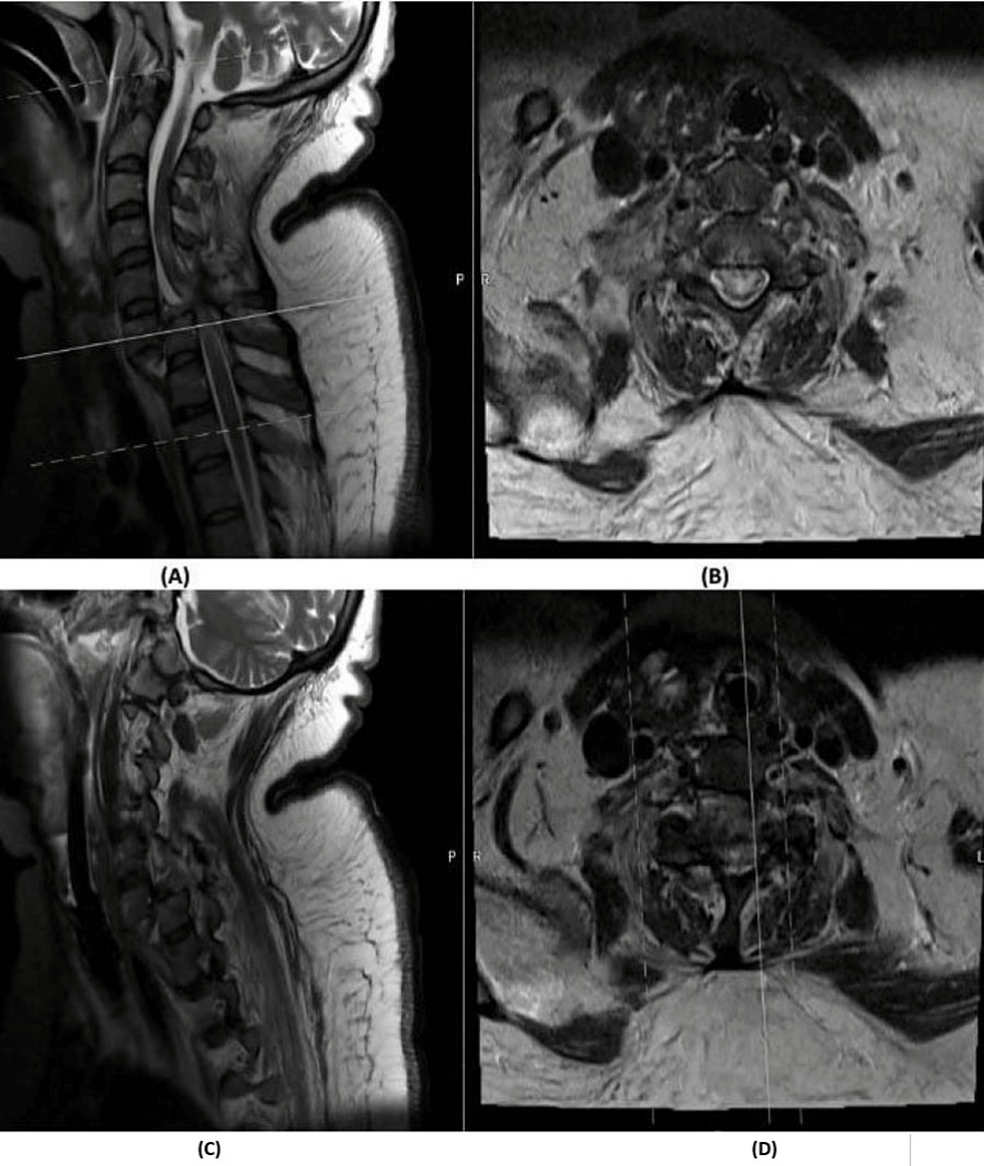
MRI of C6-C7 fracture dislocation (A,C): Sagittal spine MRI demonstrating a total fracture dislocation of C6–C7 with marked cord contusion. (B,D) Axial spine MRI of the corresponding level of C6–C7 vertebral body.

Furthermore, a sensory exam showed grade 2/2 in C5-C6 bilaterally and 0/2 from C7 to S1, while reflexes and upper motor neuron signs (i.e., Hoffman and Babinski signs) were both absent. Trauma computed tomography (CT) showed an isolated spine injury. Moreover, whole-spine CT and magnetic resonance imaging (MRI) showed isolated cervical spine injuries involving a basin tip fracture, a comminuted C1 arch fracture, a C2 fracture, and total fracture dislocation from C6 to C7 (Figures 1-3). In addition, MRI revealed cord contusion at the same level, with C1-C2 left atlantoaxial joint instability (Figure 4).

A neck magnetic resonance angiogram and carotid CT angiogram showed attenuation of the left VA and multiple but small subacute infractions in the posterior inferior cerebellar artery. The patient was admitted to the intensive care unit (ICU), where a septic workup revealed that she had a respiratory infection. A multidisciplinary team approach involving the ICU, an infectious disease team, and vascular surgery achieved proper stabilization and medical management.

Further, the patient developed a pressure ulcer in the occipital area, which was followed and treated by wound care and plastic surgery teams. The decision was made to treat the skull and C1 fractures conservatively with a C-collar because the fractures were stable, as shown in the MRI, and due to the presence of an occipital area pressure ulcer.

**Figure 5:**
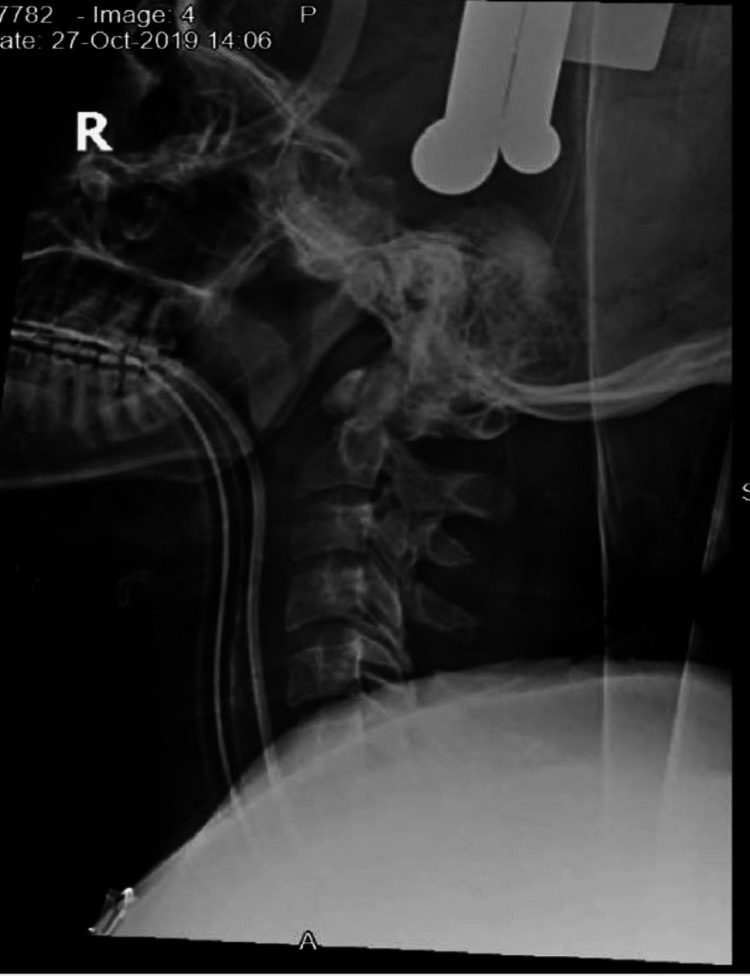
Intra-op lateral cervical X-ray Lateral cervical spine X-ray showing Gardner–Wells traction.

Due to the delayed presentation of the injury, respiratory infection, and prolonged intubation, the ICU team suggested an immediate postoperative tracheostomy. Meanwhile, gradual skeletal traction was applied using Gardner-Wells pins, reaching 20 lb for two weeks prior to surgical intervention, with some distraction of C6 from C7 achieved but not reduction (Figure 5).

The patient was cleared for surgery five weeks following the injury, with a total of two weeks of traction. The plan was to perform a single procedure using a single approach to achieve a reasonable reduction and stable cervical spine to expedite the patient’s mobility and rehabilitation. This was discussed with the patient’s family, and surgical consent was secured.

**Figure 6:**
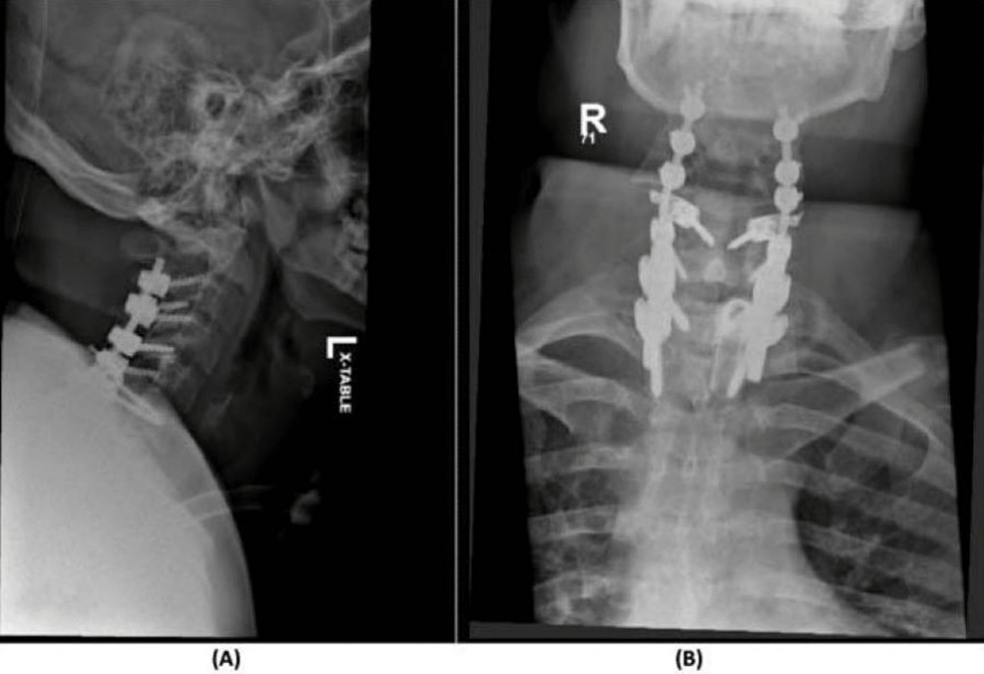
X-rays of final operative reduction and fixation (A) Lateral cervical spine X-ray demonstrating final fixation from C3–T2; (B) AP spine X-ray demonstrating the corresponding level.

**Figure 7:**
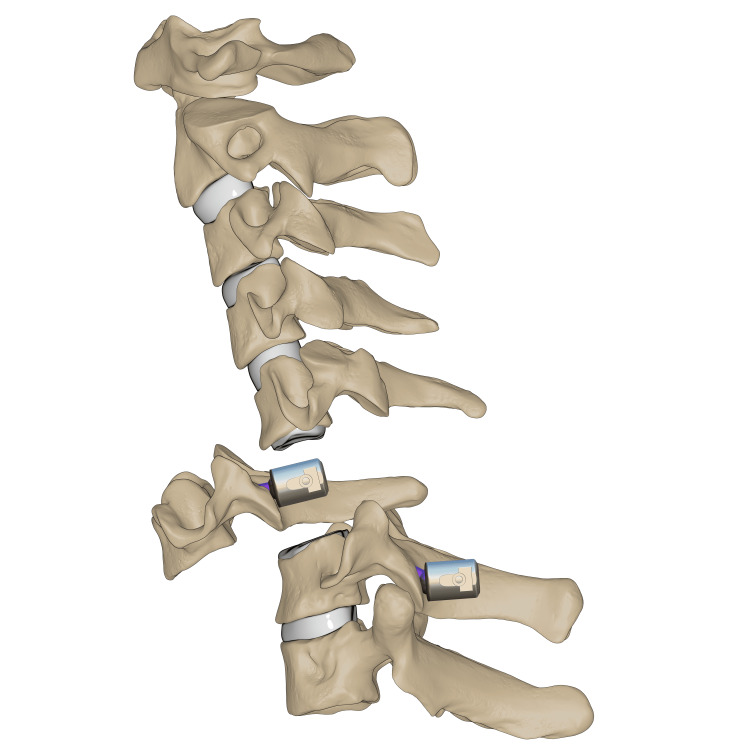
C6-7 transpedicular screw fixation Illustration of transpedicular screws fixed at C6–C7 levels. *This illustration is the authors' own creation.

General anesthesia was administered intraoperatively. After replacing the Gardner-Wells traction with a Mayfield frame, the patient was placed in a prone position on a Jackson table under an image intensifier. Manual closed reduction was attempted using Mayfield traction but was unsuccessful. The patient underwent the standard posterior approach for posterior spinal instrumentation of C3-C5 with lateral mass screws and pedicle instrumentation of C7 and T1-T2 utilizing an intraoperative X-ray, O-arm, and navigation system. A wide laminectomy and complete facetectomy at C5, C6, and C7 levels were performed. Upon direct visualization, the spinal cord was severely contused, which correlated with the MRI and clinical exam. The C6 nerve roots were fully exposed bilaterally, and it was possible to release part of the C6-7 disc from both sides of the cord posteriorly using a knife. At the level of C6, and due to the fracture pattern, which involved both C6 pedicles, the decision was made to insert a transpedicular screw at C6 (Figures 6-7). Since the right pedicle of C6 was fractured and detached from the C6 body, this screw was inserted to mimic a transpedicular screw without the presence of the pedicle. A 3.5 mm partially threaded screw (under C-arm guidance, so as not to breach anteriorly) was used, with the screw head and non-threaded part prominent from the body. Since the left-side pedicle was fractured at the superior-articular facet, transpedicular screw fixation was employed after removing the junction of the superior-articular facet using a half-threaded screw, with the head and non-threaded part prominent from the pedicle.

**Figure 8:**
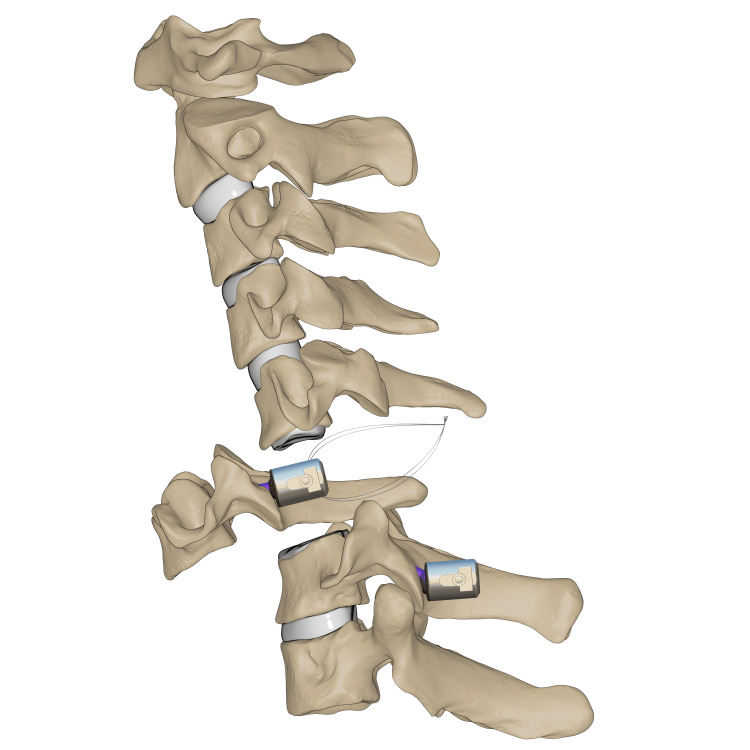
C6 sublaminar wire Illustration of sublaminar wire shown passing the C6 pedicle screw. *This illustration is the authors' own creation.

**Figure 9:**
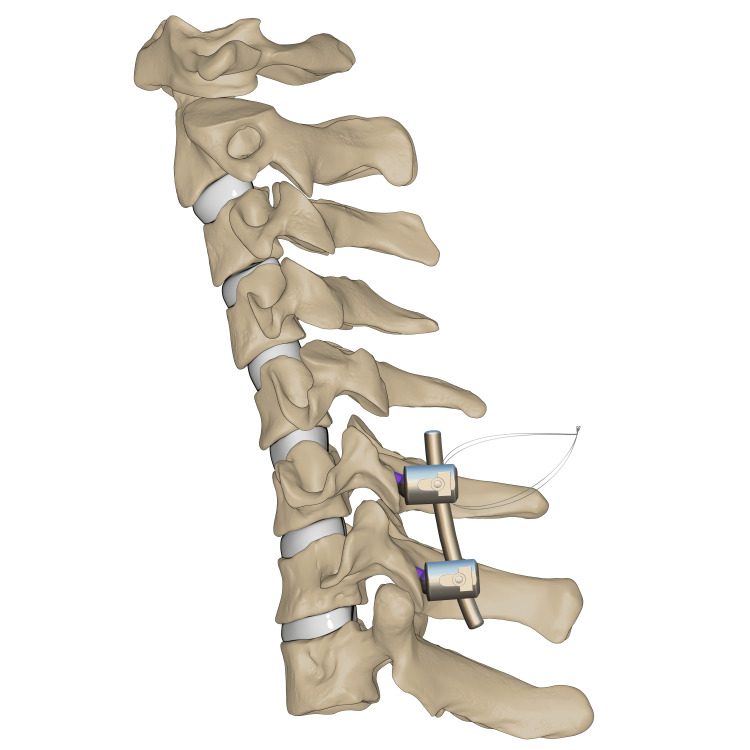
C6-7 reduction via sequential tensioning Illustration of C6–C7 reduction depicted after application of the rod followed by sequential wire tensioning. *This illustration is the authors' own creation.

**Figure 10:**
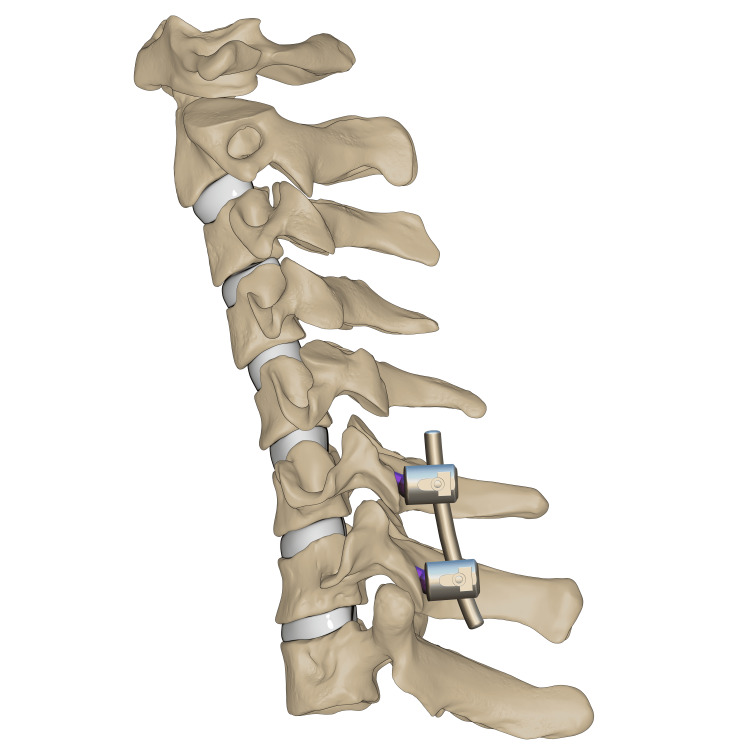
C6-7 final reduction Illustration of the final reduction and instrumentation of C6–C7 after sublaminar wire removal. *This illustration is the authors' own creation.

**Figure 11:**
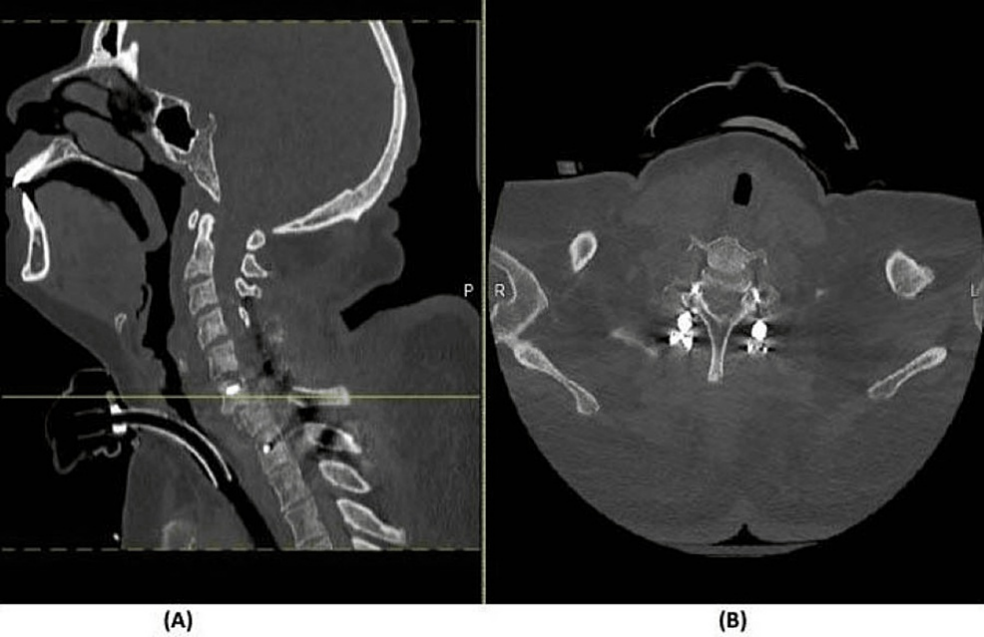
CT scan of the final C6-7 fracture reduction (A) Sagittal spine CT demonstrating near total anatomical reduction of C6–C7 fracture dislocation; (B) axial spine CT of the corresponding level of C6–C7 vertebral body.

O-arm-navigated transpedicular screw fixation was used as the C7 fixation method. This transpedicular-body screw consisted of a half-threaded screw, which would later act as a reduction mechanism (Figure 7). Almost half of the screw was left prominent, with a free screw head. Using a 3.5 mm rod connected end to end to a 5.5 mm rod, contouring for the proper cervical lordosis was done, mimicking the cervicothoracic junction. The rod was applied distally at C7, T1, and T2, and set screws (the locking mechanism) were applied and tightened. At the level of C6, the transpedicular body screw was 2 cm deep from the rod, so a sublaminar wire was passed and tightened to a set screw to connect the wire to the rod (Figures 8-9). Alternating sequential tensioning of the sublaminar wire from both sides against the rod was performed to reduce the C6-7 dislocation (Figures 10-11).

It was difficult to wean the patient off mechanical ventilation due to the prolonged intubation period. Hence, the decision was made to proceed with a tracheostomy, which enabled this to be done. Intraoperative cultures were positive for pseudomonas, and appropriate antibiotics were started promptly following consultation with the infectious disease team. The patient stayed in the ICU for 41 days and was stable enough to be transferred to the ward. At this point, her infection was controlled, and she was vitally stable. The patient failed a swallowing assessment as well as a nasogastric tube insertion, so a peg tube was inserted to optimize her nutrition.

Unfortunately, the patient did not regain any power or sensation compared to her preoperative condition. However, she was clinically stable enough, so a spinal cord injury rehabilitation program was initiated.

## Discussion

Sub-axial cervical spine injuries are often missed and may lead to devastating complications, ranging from pain to progressive neurological deficits [7]. One study reported that 4.6% of cervical spine injuries were missed upon initial evaluation in the emergency department [8]. In terms of treatment, the most common method is closed reduction, followed by anterior, posterior, or combined fixation and fusion [9-11]. Controversy exists regarding whether the closed reduction is successful for these types of delayed injuries. Srivastava et al. reported six cases of delayed cervical spine dislocations; one case was reduced preoperatively, while two were reduced under general anesthesia, resulting in a success rate of 50% [12]. Conversely, Goni et al. reported a similar number of cases, but closed reduction was not successful in any of them. Consequently, they suggested that closed reduction was not useful in cases of neglected cervical dislocation [13]. However, in the former study, the protocol consisted of skeletal traction for three weeks, starting with 5 kg and increasing to a maximum of 8-10 kg, and if the reduction failed, a trial under general anesthesia was attempted. In the latter study, the protocol only consisted of one week of skeletal traction, followed by surgical reduction and fixation if the reduction failed.

Furthermore, one study reported 10 cases of delayed cervical spine dislocation, which were treated six weeks after diagnosis [14]. Only two of the cases in that series received stabilization, the first by skull traction for a week and the second by anterior discectomy and fusion. The rest underwent posterior facetectomy, followed by another week of skull traction, and then stabilization by anterior corpectomy with discectomy and fusion. In our opinion, this is a lengthy treatment approach with multiple repositionings during surgery, which adds to the anesthesia time and may lead to complications. Nevertheless, multiple approaches are necessary in some cases to achieve anatomical reduction, as discussed by Payer et al. when explaining the anterior-posterior-anterior approach [15]. They argued that the key to a successful reduction is the removal of the fibrocartilaginous tissue around the uncovertebral and facet joints using the anterior-posterior approach.

Numerous studies have shown that fracture chronicity makes surgical treatment more challenging. The reasons for delayed diagnosis, and hence treatment, have been explained in multiple studies and include the lack of a cervical spine radiological workup due to the presence of a distracting injury (e.g., a head injury) or, in the case of a normal neurological exam and low pain expression, inadequate radiological exposure of the cervical spine, most commonly due to radiographic misinterpretation [10,14]. In our case, the delay in diagnosis was due to the patient's delayed transfer to a tertiary care hospital.

The proposed approach is more justified in delayed cases that possibly exceed one month. In the presented case, we were able to successfully reduce and fix the fracture dislocation nearly anatomically with no need for an additional approach.

## Conclusions

Cervical spine fracture dislocation is an important injury that must be diagnosed accurately and managed acutely. A delay in surgical treatment will likely result in the progression of neurological deficits or injuries and difficulty in surgical reduction, potentially requiring more than one surgical approach. The reduction can be achieved with adequate duration of preoperative traction and an isolated approach, whether anterior or posterior.
